# Supplementary material for: Low birth weight, household socio-economic status, water and sanitation are associated with stunting and wasting among children aged 6–23 months: Results from a national survey in Ghana
Source: PLoS One. 2024 Mar 28;19(3):e0297698. doi: 10.1371/journal.pone.0297698 (PMC10977686; doi:10.1371/journal.pone.0297698)
Supplement: Attachment — Submitted filename: PONE-D-23-13762.pdf [file pone.0297698.s002.pdf]

PLOS ONE

Low birth weight, household socio-economic status, water and sanitation are associated with stunting and wasting among children aged 6-23 months: results from a national survey in Ghana

--Manuscript Draft--

Manuscript Number:	PONE-D-23-13762
Article Type:	Research Article
Full Title:	Low birth weight, household socio-economic status, water and sanitation are associated with stunting and wasting among children aged 6-23 months: results from a national survey in Ghana
Short Title:	Factors associated with with stunting and wasting in Ghana
Corresponding Author:	Hammond Yaw Addae, Mphil<br>Nursing and Midwifery Training College, Kpembe<br>Kpembe, Savannah Region GHANA
Keywords:	wasting; Stunting; Infant and young child feeding practices; water and sanitation; Ghana multi-indicator cluster survey
Abstract:	ABSTRACT<br>Background: Stunting and wasting are key public health problems in Ghana that are significantly linked with mortality and morbidity risk among children. However, information on their associated factors using nationally representative data is scanty in Ghana. This study investigated the influence of Infant and Young Child Feeding (IYCF) indicators, socio-demographic and economic related factors, and water and sanitation on stunting and wasting, using nationally representative data in Ghana.<br>Methods: The 2017/18 Ghana Multi-Indicator Cluster Survey data was analyzed. The multi-indicator cluster survey is a national cross-sectional household survey with rich data on women of reproductive age and children under the age of five. The survey used a two-stage sampling method in the selection of respondents and a computer-assisted personal interviewing technique to administer structured questionnaires from October 2017 to January 2018. The present study involved 2529 mother-child pairs, with their children aged 6 to 23 months. We used the Complex Sample procedures in SPSS, adjusting for clustering and stratification effects. In a bivariate logistic regression, variables with P-values ≤ 0.5 were included in a backward multivariate logistic regression to identify the significant factors associated with stunting and wasting.<br>Results: The mean age of children was 14.32 ± 0.14 months, with slightly more being males (50.4%). About 12.1% and 15.9% of the children were wasted and stunted, respectively. There were 39.4%, 25.9%, and 13.7% of children who, respectively, satisfied the minimum meal frequency (MMF), minimum dietary diversity (MDD), and minimum acceptable diet (MAD). None of the IYCF indicators was significantly associated with stunting or wasting in the multivariate analysis but low socio-economic status, low birth weight, being a male child and unimproved toilet facilities were significantly associated with both wasting and stunting.<br>Conclusion: Our findings suggest that aside from the pre-natal period, in certain contexts, household factors such as low socio-economic status and poor water and sanitation, may be stronger predictors of undernutrition. A combination of nutrition-specific and nutrition-sensitive interventions including the pre-natal period to simultaneously address the multiple determinants of undernutrition need strengthening.
Order of Authors:	Hammond Yaw Addae, Mphil
	Sulemana Mohammed, Msc
	Rafatu Tahiru, Msc
	Tamimu Yakubu, Msc
	Ambrose Atosona, Mphil
	Fusta Azupogo

Powered by Editorial Manager® and ProduXion Manager® from Aries Systems Corporation

Additional Information:	
**Question**	**Response**
**Financial Disclosure**<br><br>Enter a financial disclosure statement that describes the sources of funding for the work included in this submission. Review the submission guidelines for detailed requirements. View published research articles from *PLOS ONE* for specific examples.<br><br>This statement is required for submission and **will appear in the published article** if the submission is accepted. Please make sure it is accurate.<br><br>**Unfunded studies**<br>Enter: *The author(s) received no specific funding for this work.*<br><br>**Funded studies**<br>Enter a statement with the following details:<br>• Initials of the authors who received each award<br>• Grant numbers awarded to each author<br>• The full name of each funder<br>• URL of each funder website<br>• Did the sponsors or funders play any role in the study design, data collection and analysis, decision to publish, or preparation of the manuscript?<br>• **NO** - Include this sentence at the end of your statement: *The funders had no role in study design, data collection and analysis, decision to publish, or preparation of the manuscript.*<br>• **YES** - Specify the role(s) played.<br><br>* typeset	The authors received no specific funding for this work.
**Competing Interests**<br><br>Use the instructions below to enter a competing interest statement for this submission. On behalf of all authors, disclose any competing interests that could be perceived to bias this work—acknowledging all financial support and any other relevant financial or non-financial competing interests.	The authors have declared that no competing interests exist.

This statement is required for submission and **will appear in the published article** if the submission is accepted. Please make sure it is accurate and that any funding sources listed in your Funding Information later in the submission form are also declared in your Financial Disclosure statement.<br><br>View published research articles from *PLOS ONE* for specific examples.<br><br>**NO authors have competing interests**<br><br>Enter: *The authors have declared that no competing interests exist.*<br><br>**Authors with competing interests**<br><br>Enter competing interest details beginning with this statement:<br><br>*I have read the journal's policy and the authors of this manuscript have the following competing interests: [insert competing interests here]*<br><br>* typeset	
**Ethics Statement**<br><br>Enter an ethics statement for this submission. This statement is required if the study involved:<br><br>• Human participants<br>• Human specimens or tissue<br>• Vertebrate animals or cephalopods<br>• Vertebrate embryos or tissues<br>• Field research<br><br>Write "N/A" if the submission does not require an ethics statement.<br><br>General guidance is provided below. Consult the submission guidelines for detailed instructions. **Make sure that all information entered here is included in the**	N/A

**Methods section of the manuscript.**<br><br>**Format for specific study types**<br><br>**Human Subject Research (involving human participants and/or tissue)**<br>• Give the name of the institutional review board or ethics committee that approved the study<br>• Include the approval number and/or a statement indicating approval of this research<br>• Indicate the form of consent obtained (written/oral) or the reason that consent was not obtained (e.g. the data were analyzed anonymously)<br><br>**Animal Research (involving vertebrate animals, embryos or tissues)**<br>• Provide the name of the Institutional Animal Care and Use Committee (IACUC) or other relevant ethics board that reviewed the study protocol, and indicate whether they approved this research or granted a formal waiver of ethical approval<br>• Include an approval number if one was obtained<br>• If the study involved *non-human primates*, add *additional details* about animal welfare and steps taken to ameliorate suffering<br>• If anesthesia, euthanasia, or any kind of animal sacrifice is part of the study, include briefly which substances and/or methods were applied<br><br>**Field Research**<br><br>Include the following details if this study involves the collection of plant, animal, or other materials from a natural setting:<br>• Field permit number<br>• Name of the institution or relevant body that granted permission	
**Data Availability**<br><br>Authors are required to make all data underlying the findings described fully available, without restriction, and from the time of publication. PLOS allows rare exceptions to address legal and ethical concerns. See the PLOS Data Policy and FAQ for detailed information.	No - some restrictions will apply

Powered by Editorial Manager® and ProduXion Manager® from Aries Systems Corporation

A Data Availability Statement describing where the data can be found is required at submission. Your answers to this question constitute the Data Availability Statement and **will be published in the article**, if accepted.<br><br>**Important:** Stating 'data available on request from the author' is not sufficient. If your data are only available upon request, select 'No' for the first question and explain your exceptional situation in the text box.<br><br>Do the authors confirm that all data underlying the findings described in their manuscript are fully available without restriction?	
**Describe where the data may be found in full sentences. If you are copying our sample text, replace any instances of XXX with the appropriate details.**<br><br>• If the data are **held or will be held in a public repository**, include URLs, accession numbers or DOIs. If this information will only be available after acceptance, indicate this by ticking the box below. For example: *All XXX files are available from the XXX database (accession number(s) XXX, XXX.).*<br>• If the data are all contained **within the manuscript and/or Supporting Information files**, enter the following: *All relevant data are within the manuscript and its Supporting Information files.*<br>• If neither of these applies but you are able to provide **details of access elsewhere**, with or without limitations, please do so. For example:<br><br>*Data cannot be shared publicly because of [XXX]. Data are available from the XXX Institutional Data Access / Ethics Committee (contact via XXX) for researchers who meet the criteria for access to confidential data.*<br><br>*The data underlying the results*	We do not have the right to distribute the datasets used for our analysis. Datasets can be accessed on copyright holders' website https://mics.unicef.org/surveys

presented in the study are available from (include the name of the third party and contact information or URL).<br>• This text is appropriate if the data are owned by a third party and authors do not have permission to share the data.<br><br>* typeset	
Additional data availability information:	

Manuscript
Click here to access/download;Manuscript;PLoS_Stunting and Wasting among Children in Ghana.docx

TITLE:

Low birth weight, household socio-economic status, water and sanitation are associated with stunting and wasting among children aged 6-23 months: results from a national survey in Ghana

Addae Yaw Hammond[1,5], Mohammed Sulemana[1], Yakubu Tamimu[2,5], Atosona Ambrose[4,5] Tahiru Rafatu[3,5] and Azupogo Fusta[6]

Corresponding author: Addae Yaw Hammond[1,5]: addae@kpmbenmtc.edu.gh or hamondd@yahoo.com

Authors' affiliations

[1]Nursing & Midwifery Training College, Kpembe, Salaga, Ghana.

[2]Department of Nutrition & Dietetics, Tamale Technical University, Tamale, Ghana

[3]Community Health Nurse Training College, Tamale, Ghana

[4]Department of Nutritional Sciences, School of Allied Health Sciences, University for Development Studies, Tamale, Ghana

[5]Department of Biochemistry and Biotechnology, College of Science, Kwame Nkrumah University of Science and Technology, Kumasi, Ghana.

[6]Department of Family and Consumer Sciences, Faculty of Agriculture, Food and Consumer Sciences, University for Development Studies, Tamale, Ghana.

ABSTRACT

Background: Stunting and wasting are key public health problems in Ghana that are significantly linked with mortality and morbidity risk among children. However, information on their associated factors using nationally representative data is scanty in Ghana**.** This study investigated the influence of Infant and Young Child Feeding (IYCF) indicators, socio-demographic and economic related factors, and water and sanitation on stunting and wasting, using nationally representative data in Ghana.

Methods: The 2017/18 Ghana Multi-Indicator Cluster Survey data was analyzed. The multi-indicator cluster survey is a national cross-sectional household survey with rich data on women of reproductive age and children under the age of five. The survey used a two-stage sampling method in the selection of respondents and a computer-assisted personal interviewing technique to administer structured questionnaires from October 2017 to January 2018. The present study involved 2529 mother-child pairs, with their children aged 6 to 23 months. We used the Complex Sample procedures in SPSS, adjusting for clustering and stratification effects. In a bivariate logistic regression, variables with P-values ≤ 0.5 were included in a backward multivariate logistic regression to identify the significant factors associated with stunting and wasting.

Results: The mean age of children was 14.32 ± 0.14 months, with slightly more being males (50.4%). About 12.1% and 15.9% of the children were wasted and stunted, respectively. There were 39.4%, 25.9%, and 13.7% of children who, respectively, satisfied the minimum meal frequency (MMF), minimum dietary diversity (MDD), and minimum acceptable diet (MAD). None of the IYCF indicators was significantly associated with stunting or wasting in the multivariate analysis but low socio-economic status, low birth weight, being a male child and unimproved toilet facilities were significantly associated with both wasting and stunting.

Conclusion: Our findings suggest that aside from the pre-natal period, in certain contexts, household factors such as low socio-economic status and poor water and sanitation, may be stronger predictors of undernutrition. A combination of nutrition-specific and nutrition-sensitive interventions including the pre-natal period to simultaneously address the multiple determinants of undernutrition need strengthening.

Keywords; wasting; stunting; Infant and young child feeding practices; water and sanitation; Ghana multi-indicator cluster survey

INTRODUCTION

Globally, the most significant singular driver of mortality and morbidity is sub-optimal diet [1]. It is a well-known fact that undernutrition has retarding effects on all aspects of human development and is particularly profound among children of south-east Asia and Sub-Saharan Africa [2,3]. However, its causes are region-specific and complex. The World Health Organization estimates that 144 million, 47 million and 38 million children under five (5) years are stunted, wasted and overweight, respectively [4]. It is also estimated that 40% and 30% of the world's stunted and wasted children, respectively, live in Africa alone [4], with West Africa, in particular, having substantially higher rates [3]. In Ghana, however, although undernutrition rates are on a downward trajectory, regional disparities still exist [5,6], and current rates are still high relative to internationally acceptable thresholds [7,8]. Nationally representative estimates classify 18% of children under 5 years as stunted, 7% as wasted and 66% as anaemic, with 1 in every 19 children likely to die before age five (5) [6,9].

The ramifications and harm caused by undernutrition, especially during the first 1000 days in a child's life and its long-term effects on economic productivity, educational achievements and overall morbidity and mortality have been documented extensively [10–12]. In Ghana nonetheless, a myriad of research works have implicated sub-optimal Infant and Young Child Feeding (IYCF) practices [13,14]; poor wealth distribution [15]; low maternal education [13]; low birth weight [14]; and inadequate sanitary conditions [13] as plausible causes of malnutrition.

Despite these well-established epidemiology of malnutrition in Ghana, the lack of prioritization of interventions has led to interventions not yielding the desired impact [16]. The infant feeding landscape in Ghana has been described as one saddled with decreasing exclusive breastfeeding rates [17], increasing reliance on commercial infant formula/cereals [18], and extensive use of monotonous staples with low micronutrient content [19]. These have led to low prevalence of IYCF indicators such as Minimum Meal Frequency (MMF), Minimum Dietary Diversity (MDD), and Minimum Acceptable Diet (MAD). For instance, a Ghanaian national survey analysis found that 42% of children aged 6 to 23 months meet the recommended MMF, with 38% meeting the MDD and only 14% the MAD [20].

Promotion of nutrition-specific interventions which target immediate causes of malnutrition, such as sub-optimal dietary practices, under these contexts could mean improving malnutrition [21,22]. To a larger extent, it could also aid in ensuring optimal childhood growth, enhance cognitive development [10], reduce malnutrition rates and potentially protect children against opportunistic infections by strengthening their immune systems [23,24]. This assertion that improving dietary factors would inevitably improve malnutrition rates may only sometimes be true, as the link between sub-optimal diet and malnutrition could be amplified by other factors [25,26]. Besides, malnutrition itself could be caused by other factors that are not diet-related. Consequently, it is

being advocated that the addition of nutrition-sensitive interventions that target the underlying causes of malnutrition may be necessary and synergistic to nutrition-specific interventions if improved nutrition outcomes are to be achieved and sustained [27–29].

The multidimensional nature of the IYCF-undernutrition nexus suggests it could be complicated by extraneous factors peculiar to sub-Saharan Africa such as poor water sources [30], inadequate sanitary conditions [25,31], malarial infections [32], low socio-economic status [33] and socio-cultural norms and practices [34].

There is sparse literature exploring factors associated with both wasting and stunting using nationally representative data in Ghana. As a result, the purpose of this study was to investigate the relationship between IYCF indicators, socio-economic, demographic, water and sanitation factors, and stunting and wasting. This study would synthesize these factors of undernutrition and recommend approaches that may reduce malnutrition in the wake of Sustainable Development Goal 2.2, which seeks to reduce stunting and wasting in children under five (5) years to internationally acceptable levels by 2025 and end all forms of malnutrition by 2030 [35].

MATERIALS AND METHODS

Study design, site, and population

The 2017/18 Ghana Multi-Indicator Cluster Survey (MICS), from which the data for this study was based was a national cross-sectional household survey undertaken in Ghana by the Ghana Statistical Service in collaboration with stakeholders and development partners, to gather data on a wide range of indicators on the situation of children, and women and men aged 15-49 years. The present study focused on mother-child pairs, with their children aged 6 to 23 months. The survey

provides statistically sound data for policy formulation, monitoring progress and evaluation of existing programs' impact [36].

Sampling and sample size

The Ghana MICS 2017/18 used a two-stage sampling method which comprised a selection of 660 enumeration areas using probability proportionate to household size. The second-stage sampling employed a systematic random sampling to select 20 households in each Electoral Area (EA), resulting in 13,200 households across all ten (10) regions of Ghana. The total number of individuals in all the selected households for the Ghana MICS 2017/18 survey was 60,581, and those eligible for interview were 37,951. Out of these, 8,903 were aged 0 to 5 years. Specific details of the sampling methods have been described elsewhere [36]. Upon excluding non-response, children aged 24 months to 5 years, children less than six (6) months and those with missing anthropometry (age, weight, and height), 2,529 infants aged 6 to 23 months, each paired with their mother, remained for inclusion in the present study. The study included only 6 to 23 months children partly because the IYCF indicators, which are key aspects of this study, are only applicable among children within this age group. The flow chart showing the detailed selection of the population for analysis is shown in figure 1.

Fig 1: Flow chart showing Selection of children 6 to 23 months from MICS 2017/18

Study variables

Dependent variables

The dependent variables were stunting and wasting.

Independent variables

The independent variables were MDD, MMF, MAD, age of child, sex of child, mother's age, mother's education, place of residence, child history of fever in the past 2 weeks, timely prenatal care, child's birth weight, sex of household head, wealth index, household source of drinking water and type of toilet facility.

Data collection

The survey (MICS 2017/18) used a computer-assisted personal interviewing technique to administer pre-tested structured questionnaires to eligible respondents spanning October 2017 to January 2018. The survey employed six questionnaires: household questionnaire, water quality testing questionnaire, men's questionnaire, women's questionnaire, under-5 children's questionnaire and 5-17 years children's questionnaire. In the present study, data gathered from the water and sanitation and household characteristics modules of the household questionnaire, women's background module of the women's questionnaire and child's background, anthropometry and breastfeeding and dietary intake modules of the under-5 children's questionnaire were used. Details of the data collection methods have been published elsewhere [36]. The WHO IYCF indicators were validated for children aged 6-23 months, which is why we limited the current investigation to this age group.

Assessment of stunting and wasting

Stunting and wasting are considered the two most common indices for defining undernutrition [37]. This study used a combination of children's height, weight, sex, and age to produce anthropometric indices. Height and age were used to calculate height-for-age z-scores (HAZ), which connotes stunting status, a measure of chronic malnutrition. Weight and height were also used to calculate weight-for-height z-scores (WHZ), which reflect wasting status, a measure of acute malnutrition. Chronic malnutrition is often due to prolonged inadequate dietary intake and illness, while acute malnutrition is usually due to food deprivation and illness of recent origin [38]. Z-scores less than -2 SD relative to the 2007 WHO reference population median value [39] were considered stunted and wasted for HAZ and WHZ, respectively. And children with HAZ and WHZ equal to or more than -2 SD were considered not stunted and not wasted, respectively.

Assessment of Infant and young child feeding indicators

These indicators were measured using the 24-hour dietary recall method, and they included MDD, MMF and MAD [40]. These indicators have been validated within the developing country context as reliable indicators for predicting access to food, overall quality of diet and nutritional status of children aged 6 to 23 months [41].

To measure these indicators, this study categorised the specific foods consumed by children over the past 24 hours to conform with the WHO 8-food group indicator [40]. These comprised (1) grains, tubers, and roots, (2) dairy products, (3) meat and fish, (4) eggs (5) legumes, nuts, and seeds, (6) dark green leafy vegetables and vitamin A-rich foods, (7) other fruits and vegetables and (8) breastmilk. The operational definitions of IYCF indicators were adopted from WHO/UNICEF [40].

MDD is a WHO-recommended indicator for measuring diet quality. Children aged 6 to 23 months are deemed to have met the MDD if they ate foods from five (5) or more food groups in the past

24 hours. MMF is an age-specific indicator that measures food quantity and frequency. Regarding breastfed children, those aged 6 to 8 months who have consumed two or more solid, semi-solid, or soft feeds are deemed to have met the MMF, while those aged 9 to 23 months need three (3) or more solid, semi-solid, or soft feeds to meet the MMF. For non-breastfed children, those aged 6 to 23 months need four (4) or more solid, semi-solid, or soft feeds to meet the MMF. Regarding MAD, children were deemed to have met this indicator if they have met both MDD and MMF.

Assessment of water and sanitation systems and wealth index

The water and sanitation systems were assessed based on the recommendations of WHO and UNICEF [42]. In the present study, piped water, public tap, borehole, protected well, protected springs, rainwater in a tank, and bottled or sachet water were classified as protected water, while, river, irrigation channel, lake, dam, pond, stream, unprotected well, or unprotected spring water were categorized as unprotected water. For sanitation, flush toilets to sewer system, flush toilets to septic tank, ventilated improved pit latrine, pour-flush latrines to sewer system, pour-flush latrines to septic tanks, composting toilets, pit latrine with slab were classified as improved facilities, while flush toilets to open drain, pit latrine without slap, bucket latrine, hanging toilet or hanging latrine was classified as unimproved facilities. Open defecation was defined as households without toilet facilities or households that defecate in the bush or fields. Wealth index was computed using the principal component analysis, where household assets were used as bases for determining wealth. Wealth index was classified as low, medium or high. Specific details of computations of wealth index of the Ghana MICS 2017/2018 are described in the published report [36].

Quality control measures

The Ghana MICS 2017/18 employed several on-field methods to reduce bias and minimize error. According to the published report [36], field workers were trained extensively on interviewing techniques and questionnaire administration and practised role-play interviews. This included five (5) days in on-field practice and one (1) day in a pilot survey to be conversant with the questionnaire administration process. Field workers responsible for taking anthropometric measurements and samples for testing were trained for an additional 14 days and six (6) more days for pilot and on-field practice. Monitoring and supervision of fieldwork were carried out by team supervisors. To ensure high standards, team supervisors observed the interviewers' skills daily and re-interviewed one household in each cluster using purposive and random sampling procedures. These are contained in the published report of MICS 2017/18 [36].

Statistical analysis

This study's statistical analysis was conducted using SPSS version 22 (SSPS Inc. Chicago, IL, USA). Descriptive statistics were used to present data on the study population's socio-demographic characteristics. Bivariate analysis was performed using chi-square ($\chi 2$) test and binary logistic regression analysis to determine the association between the dependent and independent factors. The independent factors that were significant with $p < 0.05$ in the bivariate analysis were considered for multivariable logistic regression analysis. Furthermore, given that the MICS used a multistage cluster study design which deviates from the principle of equal probability in simple random sampling, clustering and stratification were adjusted for in all analyses using SPSS complex samples module procedure. This involved creating a complex samples plan file (csplan) using strata, cluster, and sample weight variables in our combined dataset. The csplan file

served as the bases for all analyses in this study. The intent was to normalise the variance and eliminate the risks of bias towards under and over-sampled subpopulations. All analyses were carried out with 95% Confidence Interval (CI), and factors were deemed statistically significant if their p-values were less than 0.05 in both the bivariate and multivariable analyses. The model fitness test was determined based on chi-square and overall model p-value using Hosmer Lemeshow test.

Ethical consideration

The Ghana Statistical Service sought and were granted ethical approval for the survey (2017/18 MICS) from the Ghana Health Service Ethical Review Committee. Participants' verbal consent was sought. Consent was obtained from parents/caretakers of participants younger than 18 years. They were made aware of the voluntary nature of the participation, as well as the confidentiality and anonymity of the data. In so doing, datasets were anonymized and authors had no access to individual data identifiers. Participants were also made aware of their freedom to discontinue the interview at any moment, as well as their right not to respond to any or all questions.

RESULTS

Characteristics of the study population

Table 1 depicts the socio-economic and demographic characteristics of the participants. The mean ages of children and mothers were found to be 14.32 ± 0.14 months (95% CI: 14.05-14.59) and 29.37 ± 0.31 years (95% CI: 28.76-29.98), respectively. The sex of children were of equal proportion. However, a larger proportion of mothers were middle-aged (42.8%), had low educational status (60.2%), were currently married (79.7%), and were health insured (53.7%).

Regarding households of these mother-child pairs, 56.6% were classified as rural, and 41.1% had a low wealth index. Most (24.0%) of the households were in the Ashanti Region, with the Upper West Region having the least (2.3%).

Table 1: Socio-economic and demographic characteristics of study population (n=2529)

Characteristic	Frequency (n=2529)	Percentage (%)
**Age of child (months)***		
6 to 11	862	34.1
12 to 17	811	32.1
18 to 23	856	33.8
**Sex of child**		
Male	1274	50.4
Female	1255	49.6
**Mother's age (years)****		
< 25	759	30.0
25 to 34	1082	42.8
>34	688	27.2
**Mother's education**		
None (No education)	562	22.2
Low (Primary & JHS	1523	60.2
High (SHS & above)	444	17.6
**Marital status**		
Married	2016	79.7
Not Married	513	20.3
**Area of residence**		
Rural	1431	56.6
Urban	1098	43.4
**Wealth**		
Low	1038	41.1
Medium	472	18.6
High	1019	40.3
**Administrative region**		
Western	290	11.5
Central	246	9.7
Greater Accra	237	9.4

Volta	209	8.3
Eastern	286	11.3
Ashanti	608	24.0
Brong Ahafo	231	9.1
Northern	281	11.1
Upper East	82	3.2
Upper West	59	2.3
**Health insurance status**		
Yes	1359	53.7
No	1170	46.3
**Place of delivery**		
Home	465	18.4
Private hospital	344	13.6
Government hospital	1720	68.0
*Mean child age ± standard error = 14.32 ± 0.14 months (95% Confidence Interval: 14.05-14.59)		
**Mean maternal age ± standard error = 29.37 ± 0.31 years (95% Confidence Interval: 28.76-29.98)		

Food groups consumption, IYCF indicators and nutritional status of children

Consumption data for the eight (8) food groups indicates that the majority (80.1%) of children ate from group 1 (grains, tubers, and roots), with less than 46% of children eating from the remaining 6 food groups in the past 24 hours (Table 2). In all, the average number of food groups children had eaten from was 3.37 ± 0.05. For the IYCF indicators, 39.3% of the children met the age-appropriate MMF and a much lower percentage of 25.9% and 13.7% met the MDD and MAD, respectively. The prevalence of wasting and stunting among the children was 12.1% and 15.9% respectively.

Stratifying nutritional status by IYCF indicators and food groups consumed (Table 2) revealed that only MDD was statistically significant with wasting but not stunting. Children who met MDD were significantly less likely to be wasted as compared to those who did not meet MDD (9.0% vs

13.2%, p = 0.044). MMF, MDD and MAD were not significantly associated with stunting. Children that were still breastfeeding were significantly less likely to be stunted as compared to children that were not (14.1% vs 21.4%, p=0.007). Those that ate dairy products (8.4% vs 13.1%, p=0.038), eggs (6.5% vs 13.5, p=0.002), other fruits and vegetables (8.8% vs 13.8%, p=0.018) were less likely to be wasted as compared to children that did not.

Table 2: Wasting and stunting stratified by IYCF indicators and food groups consumed

Characteristic	Frequency (%)	Not wasted n (%)	Wasted n (%)	Test statistic, p-value	Not Stunted n(%)	Stunted n(%)	Test statistics, p-value
**Total**	2529(%)	2223(87.9)	306(12.1)		2126(84.1)	403(15.9)	
**Minimum meal frequency**							
Yes (>4 food groups)	995(39.3)	881(88.5)	114(11.5)	χ2=0.7, p=0.62	847(85.1)	148(14.9)	χ2=1.3, p=0.390
No (< 5 food groups)	1534(60.7)	1342(87.5)	192(12.5)		1279(83.4)	255(16.6)	
**Minimum dietary diversity**							
Yes	656(25.9)	597(91.0)	59(9.0)	**χ2=8.1, p=0.044**	546(83.2)	110(16.8)	χ2=0.6, p=0.560
No	1873(74.1)	1626(86.8)	247(13.2)		1580(84.2)	293(15.6)	
**Minimum acceptable diet**							
Yes	346(13.7)	312(90.2)	34(9.8)	χ2=1.9, p=0.32	287(82.9)	59(17.1)	χ2=0.5, p=0.620
No	2183(86.3)	1911(87.5)	272(12.5)		1839(84.2)	344(15.8)	
**Currently breastfeeding**							
Yes	1969(77.9)	1710(86.8)	259(13.2)	**χ2=10.2, p=0.03**	1686(85.6)	283(14.4)	**χ2=15.4, p=0.007**
No	560(22.1)	513(91.6)	47(8.4)		440(78.6)	120(21.4)	
**Grains, tubers and roots**							
Yes	2025(80.1)	1784(88.1)	241(11.9)	χ2=0.5, p=0.73	1683(83.1)	342(16.9)	**χ2=7.9, p=0.036**
No	504(19.9)	439(87.1)	65(12.9)		443(87.9)	61(12.1)	
**Dairy products**							
Yes	534(21.1)	489(91.6)	45(8.4)	**χ2=8.5, p=0.038**	452(84.6)	82(15.4)	χ2=0.1, p=0.790
No	1995(78.9)	1734(86.9)	261(13.1)		1674(83.9)	321(16.1)	
**Meat and fish**							
Yes	1142(45.2)	1032(90.4)	110(9.6)	χ2=12.0, p=0.06	942(82.5)	200(17.5)	χ2=4.0, p=0.180
No	1387(54.8)	1191(85.9)	196(14.1)		1184(85.4)	203(14.6)	
**Eggs**							
Yes	520(20.6)	486(93.5)	34(6.5)	**χ2=19.2, p=0.002**	450(86.5)	70(13.5)	χ2=3.2, p=0.210
No	2009(79.4)	1737(86.5)	272(13.5)		1676(83.4)	333(16.6)	
**Legumes, nuts and seeds**							
Yes	422(16.7)	381(90.3)	41(9.7)		336(79.6)	86(20.4)	

No	2107(83.3)	1842(87.4)	265(12.6)	χ2=2.9, p=0.22	1790(85.0)	317(15.0)	**χ2=7.0, p=0.034**
**Dark green leafy vegetables and vitamin A-rich foods**							
Yes	1098(43.4)	980(89.3)	118(10.7)	χ2=3.6, p=0.22	889(81.0)	209(19.0)	**χ2=13.4, p=0.008**
No	1431(56.6)	1243(86.9)	188(13.1)		1237(86.4)	194(13.6)	
**Other fruits and vegetables**							
Yes	854(33.8)	779(91.2)	75(8.8)	**χ2=13.4, p=0.018**	700(82.0)	154(18.0)	χ2=4.3, p=0.100
No	1675(66.2)	1444(86.2)	231(13.8)		1426(85.1)	249(14.9)	
All food groups (mean ± standard error) = 3.37 ± 0.05 [C.I 95% =3.27 – 3.47]							

Bivariate factors associated with wasting and stunting

In a bivariate logistic regression analysis as shown in Table 3, children that met MDD were 35% less likely to be wasted as compared to those that did not meet MDD (OR = 0.65, 95% CI: 0.43–0.99, p = 0.046). Male children were about 1.5 times more likely to be wasted (OR = 1.49, 95% CI: 0.87–2.57, p = 0.036) and 1.4 times more likely to be stunted (OR = 1.43, 95% CI: 1.06–1.94, p = 0.019) as compared to their female counterparts. As compared to children of mothers with high educational status, children of mothers with low educational status were twice likely to be wasted (OR = 2.07, 95% CI: 1.27–3.39, p = 0.004) and twice likely to be stunted (OR = 2.09, 95% CI: 1.37–3.20, p = 0.001). Children that had a fever in the last two (2) weeks were about 1.5 times more likely to be wasted (OR = 1.81, 95% CI: 1.21–2.71, p = 0.004) and 1.1 times more likely to be stunted (OR = 1.48, 95% CI: 1.05–2.07, p = 0.023), as compared to children that did not have any fever. Also, children with small birth weight were 3.3 times and 2 times more likely to be wasted (OR = 3.25, 95% CI: 1.33–7.98, p = 0.010) and stunted (OR = 2.14, 95% CI: 1.11–4.12, p = 0.023) respectively, as compared to children with large birth weight. Lack of proper sanitation was a risk factor for both wasting and stunting as children of households with unimproved toilet facilities were 2 times more likely to be wasted (OR = 2.01, 95% CI: 1.05–3.85, p = 0.035) and households with open defecation were also 2 times more likely to be stunted (OR = 2.07, 95% CI:

1.19–3.59, p = 0.010) as compared to households with improved toilet facilities. Source of drinking water was only associated with wasting but not stunting; children in households with unprotected drinking water were 2 times more likely to be wasted (OR = 1.97, 95% CI: 1.07–3.62, p = 0.029) as compared to those with protected drinking water.

Table 3: Bivariate analysis of factors associated with wasting and stunting

Variable	Wasting		Stunting	
	**Unadjusted OR (CI=95%)**	**p-value**	**Unadjusted OR (CI=95%)**	**p-value**
**Minimum dietary diversity**				
Yes (≥ 5 food groups)	0.65(0.43-0.99)	**0.046**	1.1(0.80-1.50)	0.560
No (< 5 food groups)	Ref.		Ref.	
**Minimum meal frequency**				
Yes	0.90(0.59-1.38)	0.633	0.89(0.66-1.19)	0.413
No	Ref.			
**Minimum adequate diet**				
Yes	0.77(0.46-1.30)	0.330	0.90(0.59-1.36)	0.603
No	Ref.		Ref.	
**Age of child (months)**				
6 to 11	1.49(0.87-2.57)	0.150	2.94(2.06-4.19)	**<0.001**
12 to 17	0.91(0.50-1.66)	0.770	1.77(1.22-2.57)	**0.003**
18 to 23	Ref.		Ref.	
**Sex of child**				
Male	1.47(1.03-2.10)	**0.036**	1.43(1.06-1.94)	**0.019**
Female	Ref.		Ref.	
**Mother's education**				
None (No education)	2.07(1.27-3.39)	**0.004**	2.09(1.37-3.20)	**0.001**
Low (Primary & JHS)	1.46(0.86-2.48)	0.160	1.19(0.79-1.78)	0.410
High (SHS and above)	Ref.		Ref.	
**Area of residence**				
Rural	1.05(0.71-1.53)	0.820	1.39(1.01-1.90)	**0.043**
Urban	Ref.		Ref.	
**Wealth**				

Low	1.56(1.02-2.38)	**0.039**	1.49(1.05-2.12)	**0.025**
Medium	0.80(0.45-1.40)	0.431	0.99(0.63-1.55)	0.958
High	Ref.			
**Child ill with fever in last 2 weeks**				
Yes	1.81(1.21-2.71)	**0.004**	1.48(1.05-2.07)	**0.023**
No	Ref.		Ref.	
**Timely prenatal**				
Yes (< 4 months)	Ref.		Ref.	
No (4 months +)	1.91(1.04-3.50)	**0.037**	1.07(0.69-1.66)	0.774
**Child's birth weight**				
Small	3.25(1.33-7.98)	**0.010**	2.14(1.11-4.12)	**0.023**
Average	1.78(0.82-3.87)	0.144	1.20(0.75-1.91)	0.448
Large	Ref.		Ref.	
**Source of drinking water**				
Unprotected	1.97(1.07-3.62)	**0.029**	1.80(0.94-3.42)	0.074
Protected	Ref.		Ref.	
**Type of toilet facility**				
Open defecation	1.34(0.74-2.41)	0.329	2.07(1.19-3.59)	**0.010**
Unimproved	2.01(1.05-3.85)	**0.035**	0.91(0.52-1.62)	0.759
Improved	Ref.		Ref.	
**Sex of household head**				
Male	Ref.		Ref.	
Female	1.39(0.59-3.28)	0.451	0.58(0.35-0.94)	**0.026**
Ref. = Reference category				

Factors associated with wasting from multivariate analysis

The multivariable logistic regression analysis (Table 4) showed that MDD was not significantly associated with wasting. Mothers with low educational status were 2.8 times more likely to have children that were wasted than children of mothers with high educational status (AOR = 2.8, 95% CI: 1.16–6.77, p = 0.023). Further, as compared to female children, male children were 2.2 times more likely to be wasted (AOR = 2.19, 95% CI: 1.21–3.96, p = 0.009), and children that fell sick in the past 2 weeks were also 2.2 times more likely to be wasted (AOR = 2.15, 95% CI: 1.18–3.91, p = 0.012) relative to those that did not. Children with low and medium birth weight were about 5

times and 3 times more likely to be wasted respectively as compared to children with high birth weight (AOR = 4.84, 95% CI: 2.03-11.53, $p < 0.001$) and (AOR = 3.11, 95% CI: 1.74–5.58, $p < 0.001$). Lastly, children of households with unprotected sources of drinking water (AOR = 2.55, 95% CI: 1.22–5.34, p = 0.013) and unimproved toilet facilities (AOR = 2.16, 95% CI: 1.03–4.55, p = 0.042) were both about 2.5 times more likely to be wasted as compared to those that had protected source of drinking water and improved toilet facilities, respectively. Overall, these exposure factors accounted for 20.0% (Nagelkerke R^2 =0.200) of the variance in wasting among 6 to 23 months children in Ghana.

Table 4: Factors associated with wasting among children aged 6 to 23 months

Variable	Wasting			
	Adjusted OR (AOR)	95% Confidence Interval		p-value
		Lower	Upper	
**Sex of child**				
Male	2.19	1.21	3.96	**0.009**
Female	Ref.			
**Mother's education**				
None (No education)	1.94	0.66	5.70	0.230
Low (Primary & JHS)	2.80	1.16	6.77	**0.023**
High (SHS and above)	Ref.			
**Wealth**				
Low	Ref.			
Medium	0.32	0.13	0.81	**0.016**
High	0.56	0.23	1.36	0.201
**Child ill with fever in last two (2) weeks**				
Yes	2.15	1.18	3.91	**0.012**
No	Ref.			
**Child's birth weight**				
Small	4.84	2.03	11.53	**<0.001**
Average	3.11	1.74	5.58	**<0.001**
Large	Ref.			
**Source of drinking water**				
Unprotected	2.55	1.22	5.34	**0.013**
Protected	Ref.			

**Type of toilet facility**				
Open defecation	1.61	0.60	4.37	0.346
Unimproved	2.16	1.03	4.55	**0.042**
Improved	Ref.			
Ref. =Reference category; JHS=Junior High School; SHS= Senior High School; OR= Odds Ratio				
Nagelkerke R square = 0.200				

Factors associated with stunting from multivariate analysis

As shown in Table 5, male children were about 1.8 times more likely to be stunted as compared to their female counterparts (AOR = 1.80, 95% CI: 1.28–2.52, p = 0.001) and children from low wealth households were also about 2.2 times more likely to be stunted as compared to children from high wealth households (AOR = 2.18, 95% CI: 1.28–3.76, p = 0.005). Children with small birth weight were 1.6 times more likely to be stunted relative to children with high birth weight (AOR = 1.59, 95% CI: 1.11–2.27, p = 0.011). Our results also showed that children from households with improved toilet facilities were 43% less likely to be stunted as compared to children from households that practice open defecation (AOR = 0.57, 95% CI: 0.37– 0.88, p = 0.011).

Table 5: Factors associated with stunting among children aged 6 to 23 months

**Variable**	**Stunting**			
	**Adjusted OR (AOR)**	**95% Confidence Interval**		**p-value**
		**Lower**	**Upper**	
**Child's age**				
6 to 11	Ref.			

12 to 17	1.26	0.78	2.04	0.348
18 to 23	3.01	1.97	4.59	**< 0.001**
**Child's sex**				
Male	1.80	1.28	2.52	**0.001**
Female	Ref.			
**Wealth**				
Low	2.18	1.27	3.76	**0.005**
Medium	2.09	1.20	3.62	**0.009**
High	Ref.			
**Child's birth weight**				
Small	1.59	1.11	2.27	**0.011**
Average	2.97	1.72	5.15	**< 0.001**
Large	Ref.			
**Type of toilet facility**				
Open defecation	Ref.			
Unimproved	0.99	0.60	1.64	0.984
Improved	0.57	0.37	0.88	**0.011**
**Household head**				
Male	1.48	1.00	2.19	**0.049**
Female	Ref.			
Ref. =Reference category; OR = Odds Ratio				
Nagelkerke R square = 0.14				

Factors associated with both wasting and stunting among children aged 6-23 months in Ghana

As shown in figure 2, the factors associated with both wasting and stunting include being a male child, low wealth index, low birth weight and unimproved toilet facility. However, children with fever in the past 2 weeks, children of mothers with low education and children in households with unprotected source of drinking water are factors associated with only wasting.

Figure 2: Factors associated with wasting and stunting among children aged 6-23 months in Ghana

DISCUSSION

This study investigated the influence of IYCF indicators, socio-demographic and economic-related factors, and water and sanitation on stunting and wasting, using nationally representative datasets from the most recent MICS in Ghana. A total of 2,529 children aged 6 to 23 months met the inclusion criteria for the study, of which 12.1% were wasted and 15.9% stunted. These malnutrition rates are lower than that of other developing countries [43,44] but higher than the WHO minimum cut-off of 5% and 10%, respectively [38]. These high prevalence of malnutrition could be due to factors such as low economic status; inadequate water and sanitation conditions; low maternal literacy rates; fever resulting from opportunistic infections; and low birth weight; factors that are consistent with the findings of this study and other studies [45,46]. This study revealed that socio-demographic and economic factors such as wealth, child's sex, and birth weight as well as unimproved sanitary conditions were associated with both wasting and stunting.

The IYCF indicators including MMF, MDD and MAD, with prevalence rates of 39.4%, 25.9% and 13.7%, respectively, did not have significant associations with either stunting or wasting at the multivariable level. Their low prevalence rates in the present study are comparable to findings of recent studies in the same location [20,25], suggesting a need for improvement in IYCF practices, with a focus on the critical first 1000 days, in Ghana. Stunting reflects malnutrition of long-term origin, and these dietary factors were measured using 24-hour recall; hence it is plausible, as in some studies [19,47], that recent dietary factors may not be sensitive in predicting

stunting. In this study, the main IYCF indicators were not associated with stunting, even at the bivariate level. A situation which suggests recent food intake in the past 24 hours may not necessarily reflect long-term consumption patterns [48]. The lack of association between stunting and MDD has been reported in other studies [47,49-51]. Although wasting is defined as a measure of malnutrition of recent onset, this study found a weak association between MDD and wasting at the bivariate level; the observed association did not remain when adjusting for other factors including child sex and birth weight, maternal education, water and sanitation, and household wealth index. It may be that MDD is less effective in determining undernutrition in contexts with low socio-economic status, and poor water and sanitary conditions with high rates of infections. In our study, the mean MDD score (3.37 ± 0.05) was well below the recommended threshold of ≥ 5 out of the 8 food groups [40]. This could also imply that dietary inadequacies are more likely in our study population, but we could not ascertain that with the available data. Nevertheless, several studies have shown multiple dietary inadequacies among children aged 6-23 months in Ghana [52]. Dietary inadequacies have a negative implication for proper immune-system function in preventing and ensuring recovery from illness. The preceding together with poor water and sanitation partly explains the significant infection (fever) among the children in the last 2 weeks in our study. However, our finding contradicts a multi-country intervention study in Zimbabwe, Kenya and Bangladesh [53], where IYCF but not Water Sanitation and Hygiene (WASH) reduced the odds of stunting among children. That study concluded that combining IYCF interventions with WASH interventions would not reduce stunting more than implementing only IYCF. Conversely, a study in India [54] reported that interventions that combined both IYCF and sanitation would yield greater impact than each in isolation; this is consistent with findings published in the LANCET [29] where achieving 100% coverage of all the 10 IYCF interventions

will only lead to 20% reduction in undernutrition and hence emphasizes the importance of combining both IYCF and WASH interventions. Highlighting the relevance of inter-sectoral collaboration if significant progress is to be made in reducing the vicious cycle of malnutrition.

Research works have shown that lack of adequate dietary intake alone cannot explain the global burden of malnutrition [55], and diet-inclined interventions alone may not be the panacea to resolving this global malnutrition challenge [29]. Inadequate sanitary conditions and poor access to water have been recognized as increasingly complex determinants of malnutrition among children [56,57]. Among a significant proportion of Ghana's population, access to improved sanitation and protected water facilities is a challenge that may influence nutrition through several pathways. Three of such pathways have been postulated [58]; (1) diarrheal diseases - where the ingestion of pathogen-ridden human excreta from food/water inhibits gastrointestinal nutrient absorption that increases the susceptibility to opportunistic infections that are associated with diarrhoea, (2) environmental enteropathy - a sub-clinical gastrointestinal tract inflammatory condition caused by ingestion of faecal bacteria, which results in impaired intestinal immune function and increased intestinal permeability, and subsequent malabsorption of nutrients, and (3) nematode infections – as humans consume soil contaminated with worm eggs infested human excreta, these soil-transmitted helminth infestations, compete with their human host for scarce nutrients which results in growth retardation. As such, emphasis on improving access to water and good sanitary practices among children up to 24 months may help reduce the high malnutrition rates in Ghana.

A comprehensive approach that centres on access to healthcare services, enhanced pre- and post-natal outreach services in hard-to-reach communities, healthy food options, and improved water and sanitation infrastructure, are necessary to address these public health complications. By

addressing these issues, stakeholders can promote healthy pregnancies, improve maternal and young child health outcomes, and ultimately, improve overall health and well-being.

Strengths and Limitations

The main limitation of this study is its inability to infer causality of its findings due to the inherent limitation of cross-sectional studies. Some of the variables included in this study were subjectively measured and could either be subject to recall bias or measurement error. For instance, the child's birth weight was based on mother's recall which may not be a precise method for determining a child's birth weight. Additionally, the IYCF indicators relied on mothers/caregivers to recall all foods consumed in the past 24 hours, which could also suffer from recall bias. However, the effects of such bias on the outcome variables could be minimal as we expect these biases in exposure variables to be equally distributed among exposed and non-exposed respondents, given the very large sample size. The fact that the explanatory variables accounted for 20% and 14% of the outcome variables meant that this study could not include all potential exposure variables in its analysis. A limitation attributable to the MICS not including an extensive number of nutrition-centred variables. In our analysis, robust survey analysis techniques were undertaken. Considering that the MICS data is nationally presentative, our findings can be generalized to all children aged 6-23 months in Ghana.

CONCLUSION

Wasting and stunting rates were found to be higher than WHO-recommended thresholds. Contrary to our a priori expectations, IYCF indicators were not associated with either wasting or stunting. Rather unprotected water and unimproved toilet facilities, among other factors were better

predictors of wasting. Our findings indicate that although dietary diversity is an important determinant of nutritional status, in certain contexts, other factors such as poor water and sanitation, low maternal education, wealth, fever, and child's birth weight may be better predictors of undernutrition. It is evident from our analysis that the diet-sanitation-nutrition nexus is complex and may warrant further investigations. These findings equally situate maternal education and equitable wealth creation, just as WASH, as integral tools that stakeholders could harness in achieving recommended childbirth weights and adequate nutritional status among infants and young children. Due to these findings, the combination of nutrition-specific and nutrition-sensitive interventions to simultaneously address the multiple determinants of undernutrition is recommended in the Ghanaian nutrition context.

ACKNOWLEDGEMENTS

The Ghana Statistical Service and UNICEF are acknowledged for sharing with us their datasets.

REFERENCES

1. WHO. Nutrition in Universal health coverage. World Heal Organ (WHO/NMH/NHD/1924) Licence CC BY-NC-SA30 IGO. 2019; 19. Available:

https://www.who.int/publications/i/item/WHO-NMH-NHD-19.24

2. Young H, Marshak A. Persistent Global Acute Malnutrition A discussion paper on the scope of the problem, its drivers, and strategies for moving forward for policy, practice, and research. a Feinstein Int Cent Publ. 2017; 55. Available: http://fic.tufts.edu/assets/FIC-Publication-Persistent-Global-Acute-Malnutrition_web_2.26s.pdf

3. Ssentongo P, Ssentongo AE, Ba DM, Ericson JE, Na M, Gao X, et al. Global, regional and national epidemiology and prevalence of child stunting, wasting and underweight in low- and middle-income countries, 2006–2018. Sci Rep. 2021;11: 1–12. doi:10.1038/s41598-021-84302-w

4. WHO. Levels and trends in child malnutrition: Key findings of the 2020 Edition of the Joint Child Malnutrition Estimates. Geneva WHO. 2020;24: 1–16.

5. Ecker O, Asselt J van. Food and nutrition security in transforming Ghana: a descriptive analysis of national trends and regional patterns. IFPRI - Discuss Pap. 2017; vi-pp. Available: http://ebrary.ifpri.org/utils/getfile/collection/p15738coll2/id/131319/filename/131530.pdf

6. Global Nutrition Report. Global Nutrition Report | Country Nutrition Profiles - Global Nutrition Report. Global Nutrition Report. 2020. Available: https://globalnutritionreport.org/resources/nutrition-profiles/africa/western-africa/ghana/#overview

7. World Health Organization. Nutrition Landscape Information System (NLiS) country profile indicators: interpretation guide, second edition. 2nd ed. Nutrition landscape information system (NLIS) Country Profile. Geneva; 2019. Available: https://apps.who.int/iris/handle/10665/332223

8. Lois A, Emmanuel G, Melissa C, Lora I. Food Insecurity, Malnutrition, and Child Developmental and Behavioral Outcomes in Ghana. 1st ed. Bahar FMSOS, McKay MM, editors. Child Behavioral Health in Sub-Saharan Africa. Washington: Springer; 2022. doi:10.1007/978-3-030-83707-5

9. USAID. Ghana: Nutrition Profile (Updated December 2021). Nutr Profile. 2021;1: 1–8.

10. Martorell R. Improved nutrition in the first 1000 days and adult human capital and health. Am J Hum Biol. 2017;29: 1–24. doi:10.1002/ajhb.22952

11. UNICEF. First 1000 days: The critical window to ensure that children survive and thrive. Unicef.

2017; 1–3. Available: https://www.unicef.org/southafrica/SAF_brief_1000days.pdf

12. Walker JL, Littlewood R. Pragmatic implementation studies to improve nutrition practices and policies: Childcare during the first 1000 days as a contributor to long-term health. Public Health Nutr. 2018;21: 1209–1211. doi:10.1017/S1368980018000022

13. Frempong RB, Annim SK. Dietary diversity and child malnutrition in Ghana. Heliyon. 2017;3: 1–21. doi:10.1016/j.heliyon.2017.e00298

14. Boah M, Azupogo F, Amporfro DA, Abada LA. The epidemiology of undernutrition and its determinants in children under five years in Ghana. PLoS One. 2019;14: 1–23. doi:10.1371/journal.pone.0219665

15. Van De Poel E, Hosseinpoor AR, Jehu-Appiah C, Vega J, Speybroeck N. Malnutrition and the disproportional burden on the poor: The case of Ghana. Int J Equity Health. 2007;6: 1–12. doi:10.1186/1475-9276-6-21

16. Yawson AE, Amoaful EO, Senaya LK, Yawson AO, Aboagye PK, Mahama AB, et al. The lancet series nutritional interventions in Ghana: A determinants analysis approach to inform nutrition strategic planning. BMC Nutr. 2017;3: 1–8. doi:10.1186/s40795-017-0147-1

17. Mohammed S, Oakley LL, Marston M, Glynn JR, Calvert C. Time trends in the prevalence and appropriate breast determinants of age- - feeding among children aged 0 – 23 months in Ghana : a pooled analysis of based surveys , 2003 – 2017. BMJ Open. 2022;12: 1–2. doi:10.1136/bmjopen-2021-059928

18. Abizari A, Ali Z, Essah CN, Agyeiwaa P, Amaniampong M. Use of commercial infant cereals as complementary food in infants and young children in Ghana. BMC Nutr. 2017;3: 1–8. doi:10.1186/s40795-017-0191-x

19. Anin S kofi, Saaka M, Fischer F, Alexander K. Association between Infant and Young Child Feeding (IYCF) Indicators and the Nutritional Status of. MDPI Nutr. 2020;12: 1–19. doi:doi:10.3390/nu12092565

20. Donkor WES, Adu-afarwuah S, Wegmüller R, Bentil H, Petry N, Rohner F, et al. Complementary Feeding Indicators in Relation to Micronutrient Status of Ghanaian Children Aged 6 – 23 Months : Results from a National Survey. MDPI Life. 2021;11: 1–17. doi:https://doi.org/10.3390/ life11090969

21. Eileen K, Jennifer S, Meghan K, Sibhatu B. Impact of Social and Behavior Change Communication in Nutrition Specific Interventions on Selected Indicators of Nutritional Status. J Hum Nutr. 2018;2: 34–46. doi:10.36959/487/280

22. Khamis AG, Mwanri AW, Ntwenya JE, Kreppel K. The influence of dietary diversity on the nutritional status of children between 6 and 23 months of age in Tanzania. BMC Pediatr. 2019;19: 1–9. doi:10.1186/s12887-019-1897-5

23. Gruszfeld D, Socha P. Early nutrition and health: Short-and long-term outcomes. World Rev Nutr Diet. 2013;108: 32–39. doi:10.1159/000351482

24. Caroline C, Philip C. C, Elizabeth A. M. Diet and immune function. Nutrients. 2019;11: 1–9. doi:10.3390/nu11081933

25. Saaka M, Saapiire FN, Dogoli RN. Independent and joint contribution of inappropriate complementary feeding and poor water, sanitation and hygiene (WASH) practices to stunted child growth. J Nutr Sci. 2021;10: 1–10. doi:10.1017/jns.2021.103

26. UNICEF. UNICEF East Asia and Pacific Regional Office (EAPRO) Guide for Practical Joint Actions Nutrition -WASH Toolkit. 1st ed. Bangkok; 2016. Available: https://www.unicef.org/eapro/WASH_Nutrition_Toolkit_EAPRO_Final_w_ISBN_web_version_7Nov2016.pdf

27. Ruel MT, Alderman H. Nutrition-sensitive interventions and programmes: How can they help to accelerate progress in improving maternal and child nutrition? Lancet. 2013;382: 536–551. doi:10.1016/S0140-6736(13)60843-0

28. Teague J. Exploring a Comprehensive Approach to Nutrition Through Nutrition-Specific and Nutrition-Sensitive Investments. Washington; 2017. Report No.: 32. Available: https://www.bread.org/sites/default/files/downloads/briefing-paper-32-nutrition-specific-investments-april-2017.pdf

29. Black RE, Victora CG, Walker SP, Bhutta ZA, Christian P, De Onis M, et al. Maternal and child undernutrition and overweight in low-income and middle-income countries. Lancet. 2013;382: 427–451. doi:10.1016/S0140-6736(13)60937-X

30. Mabhaudhi T, Chibarabada T, Modi A. Water-Food-Nutrition-Health Nexus: Linking water to improving food, nutrition and health in Sub-Saharan Africa. Int J Environ Res Public Health.

2016;13. doi:10.3390/ijerph13010107

31. Ngure FM, Reid BM, Humphrey JH, Mbuya MN, Pelto G, Stoltzfus RJ. Water, sanitation, and hygiene (WASH), environmental enteropathy, nutrition, and early child development: Making the links. Ann N Y Acad Sci. 2014;1308: 118–128. doi:10.1111/nyas.12330

32. Ehrhardt S, Burchard GO, Mantel C, Cramer JP, Kaiser S, Kubo M, et al. Malaria, anemia, and malnutrition in African children - Defining intervention priorities. J Infect Dis. 2006;194: 108–114. doi:10.1086/504688

33. Obasohan PE, Walters SJ, Jacques R, Khatab K. Risk factors associated with malnutrition among children under-five years in sub-saharan african countries: A scoping review. Int J Environ Res Public Health. 2020;17: 1–24. doi:10.3390/ijerph17238782

34. Bain LE, Awah PK, Geraldine N, Kindong NP, Sigal Y, Bernard N, et al. Malnutrition in Sub - Saharan Africa: Burden, causes and prospects. Pan Afr Med J. 2013;15: 1–9. doi:10.11604/pamj.2013.15.120.2535

35. Osborn D, Cutter A, Ullah F. Universal Sustainable Development Goals: Understanding the transformational challenge for developed countries. Univers Sustain Dev Goals. New York City; 2015. Available: https://sustainabledevelopment.un.org/content/documents/1684SF_-_SDG_Universality_Report_-_May_2015.pdf

36. Ghana Statistical Service. Multiple Indicator Cluster Survey (MICS) 2018, Survey Findings Report. Mult Indic Clust Surv (MICS 2017/18). Accra, Ghana; 2018. Available: https://statsghana.gov.gh/gssmain/fileUpload/pressrelease/MICS SFR final_compressed.pdf

37. Reinhard I, Wijayaratne KBS. The Use of Stunting and Wasting as Indicators for Food Insecurity and Poverty. Integr Foof Secur Program Trincomalee. 2000;27: 1–15. Available: http://www.sas.upenn.edu/~dludden/stunting-wasting.pdf

38. World Health Organization. Interpretation Guide Nutrition Landscape Information System (NLIS). WHO. Geneva; 2010. doi:10.1159/000362780.Interpretation

39. WHO. WHO Child Growth Standards. WHO Libr Press. Geneva; 2006. doi:10.1111/j.1469-8749.2009.03503.x

40. WHO/UNICEF. Indicators for assessing infant and young child feeding practices; Definitions and measurement methods. WHO/UNICEF Tech Rep Ser. Geneva; 2021. Available: https://apps.who.int/iris/rest/bitstreams/1341846/retrieve

41. FANTA. Working Group on Infant and Young Child Feeding Indicators. Dev Validating Simple Indic Diet Qual Infants Young Child Dev Ctries Addit Anal 10 Data Sets. Washington; 2007. Available: https://www.fantaproject.org/sites/default/files/resources/IYCF_Datasets_2007.pdf

42. WHO/UNICEF. Progress on Sanitation and Drinking water - methodology 2015 update & SDG baselines. Geneva; 2018. Available: https://washdata.org/sites/default/files/documents/reports/2018-04/JMP-2017-update-methodology.pdf

43. Ahmadi D, Amarnani E, Sen A, Ebadi N, Cortbaoui P, Melgar-Quiñonez H. Determinants of child anthropometric indicators in Ethiopia. BMC Public Health. 2018;18: 1–9. doi:10.1186/s12889-018-5541-3

44. Woodruff BA, Wirth JP, Ngnie-Teta I, Beaulière JM, Mamady D, Ayoya MA, et al. Determinants of Stunting, Wasting, and Anemia in Guinean Preschool-Age Children: An Analysis of DHS Data From 1999, 2005, and 2012. Food Nutr Bull. 2018;39: 39–53. doi:10.1177/0379572117743004

45. Katoch OR. Determinants of malnutrition among children: A systematic review. Nutrition. 2022;96: 111565. doi:10.1016/j.nut.2021.111565

46. Tasnim T. Determinants of malnutrition in children under five years in developing countries: A systematic review. Indian J Public Heal Res Dev. 2018;9: 333–338. doi:10.5958/0976-5506.2018.00574.0

47. Campbell RK, Aguayo VM, Kang Y, Dzed L, Joshi V, Waid J, et al. Infant and young child feeding practices and nutritional status in Bhutan. Matern Child Nutr. 2018;14: 1–6. doi:10.1111/mcn.12762

48. Virginia S, Baranowski T, Ronette B, Yvonne B, Laura C, Robert W. Dietary Risk Assessment in the WIC Program. Natl Acad Press. Washington; 2002. doi:10.17226/10342

49. Mank I, Vandormael A, Traoré I, Ouédraogo WA, Sauerborn R, Danquah I. Dietary habits associated with growth development of children aged < 5 years in the Nouna Health and Demographic Surveillance System, Burkina Faso. Nutr J. 2020;19: 1–14. doi:10.1186/s12937-

020-00591-3

50. Reinbott A, Kuchenbecker J, Herrmann J, Jordan I, Muehlhoff E, Kevanna O, et al. A child feeding index is superior to WHO IYCF indicators in explaining length-for-age Z-scores of young children in rural Cambodia. Paediatr Int Child Health. 2015;35: 124–134. doi:10.1179/2046905514Y.0000000155

51. Saaka M, Wemakor A, Abizari AR, Aryee P. How well do WHO complementary feeding indicators relate to nutritional status of children aged 6-23 months in rural Northern Ghana? BMC Public Health. 2015;15: 1–12. doi:10.1186/s12889-015-2494-7

52. Brouwer ID, Jager I de, Borgonjen K, Azupogo F, Rooij M, Folson G, et al. Development of food-based dietary recommendations for children, 6-23 months old, in Karaga District and Gomoa East District, Ghana. Glob Alliance Improv Nutr. 2017; 1–127. Available: http://www.gainhealth.org/wp-content/uploads/2018/03/Ghana-Development-of-Food-based-Dietary-Recommendations-Using-Optifood-Karaga-and-Gomoa-Districts.pdf

53. Pickering AJ, Null C, Winch PJ, Mangwadu G, Arnold BF, Prendergast AJ, et al. The WASH Benefits and SHINE trials: interpretation of WASH intervention effects on linear growth and diarrhoea. Lancet Glob Heal. 2019;7: 1139–1146. doi:10.1016/S2214-109X(19)30268-2

54. Parikh P, Kwami CS, Khanna R, Lall M, Reddy H, Benton L, et al. Linkages between environmental factors (Wash and energy) and infant and young child feeding practices in rural india: Implications for cross-sectoral interventions for child health. J Water Sanit Hyg Dev. 2021;11: 902–915. doi:10.2166/washdev.2021.005

55. The Global Nutrition Report. The Burden of Malnutrition. 2018 Glob Nutr Rep. 2018; 29–51. Available: https://globalnutritionreport.org/reports/global-nutrition-report-2018/

56. SNV. Water , Sanitation and Hygiene (WASH) related determinants of under-nutrition. ENUFF Proj Learn Br no 4. Houaphanh; 2020. Available: https://snv.org/assets/explore/download/snv_enuff_learning_brief_4_-_online.pdf

57. Mills JE, Cumming O. The impact of water, sanitation and hygiene on key health and social outcomes. Sanitation and Hygiene Applied Research for Equity (SHARE) and UNICEF. Sanit Hyg Appl Res Equity UNICEF. 2016; 1–112.

58. UNICEF. The Impact of Poor Sanitation on Nutrition. Sanit Hyg Appl Res Equity. 2014;1: 1–12.

Available: https://thousanddays.org/wp-content/uploads/The-Impact-of-Poor-Sanitation-on-Nutrition-1.pdf

Fig 1

Click here to access/download;Figure;Fig 1 flow Chart diagram.jpg

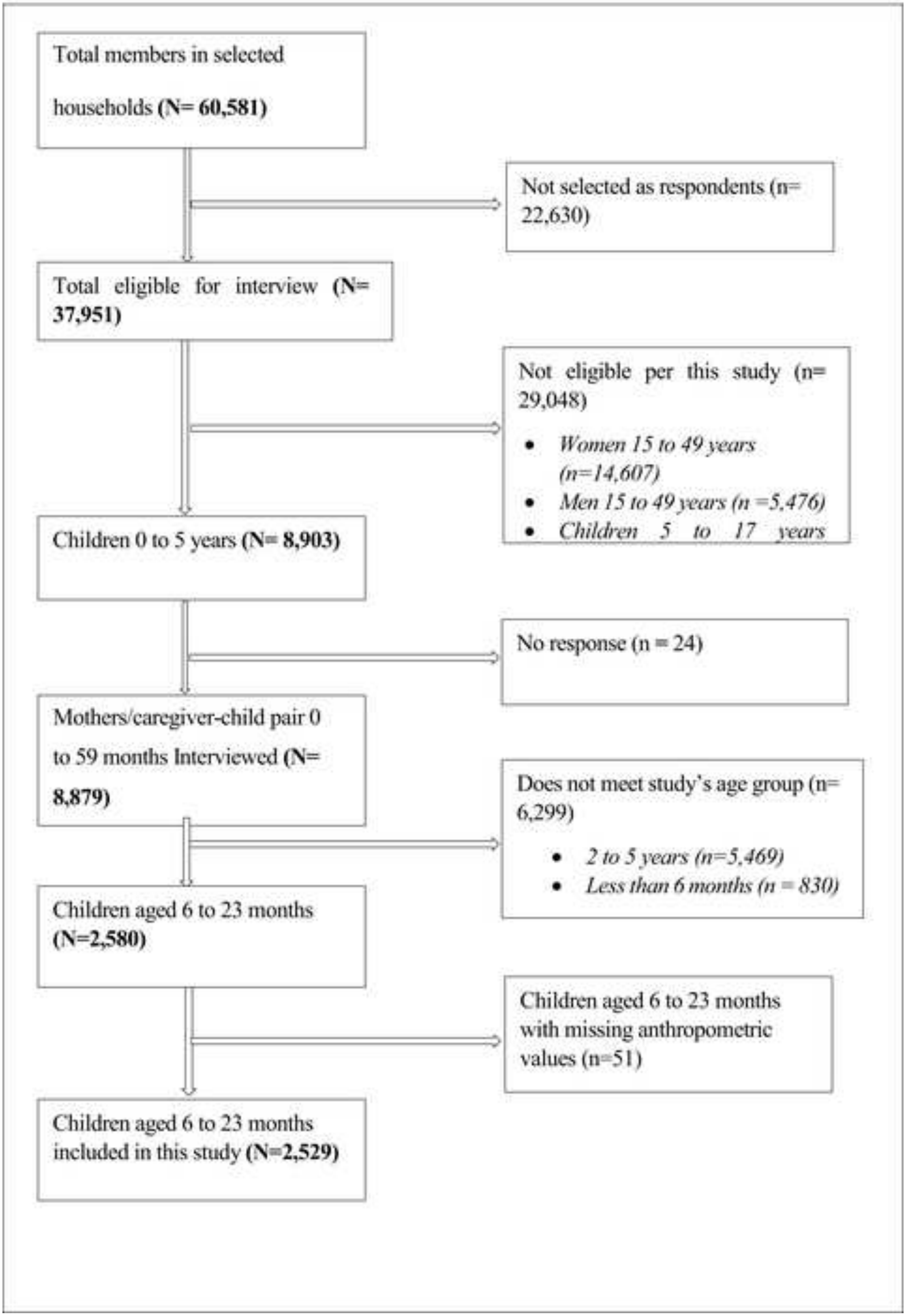

Click here to access/download;Figure;Fig 2 Factors associated.tif

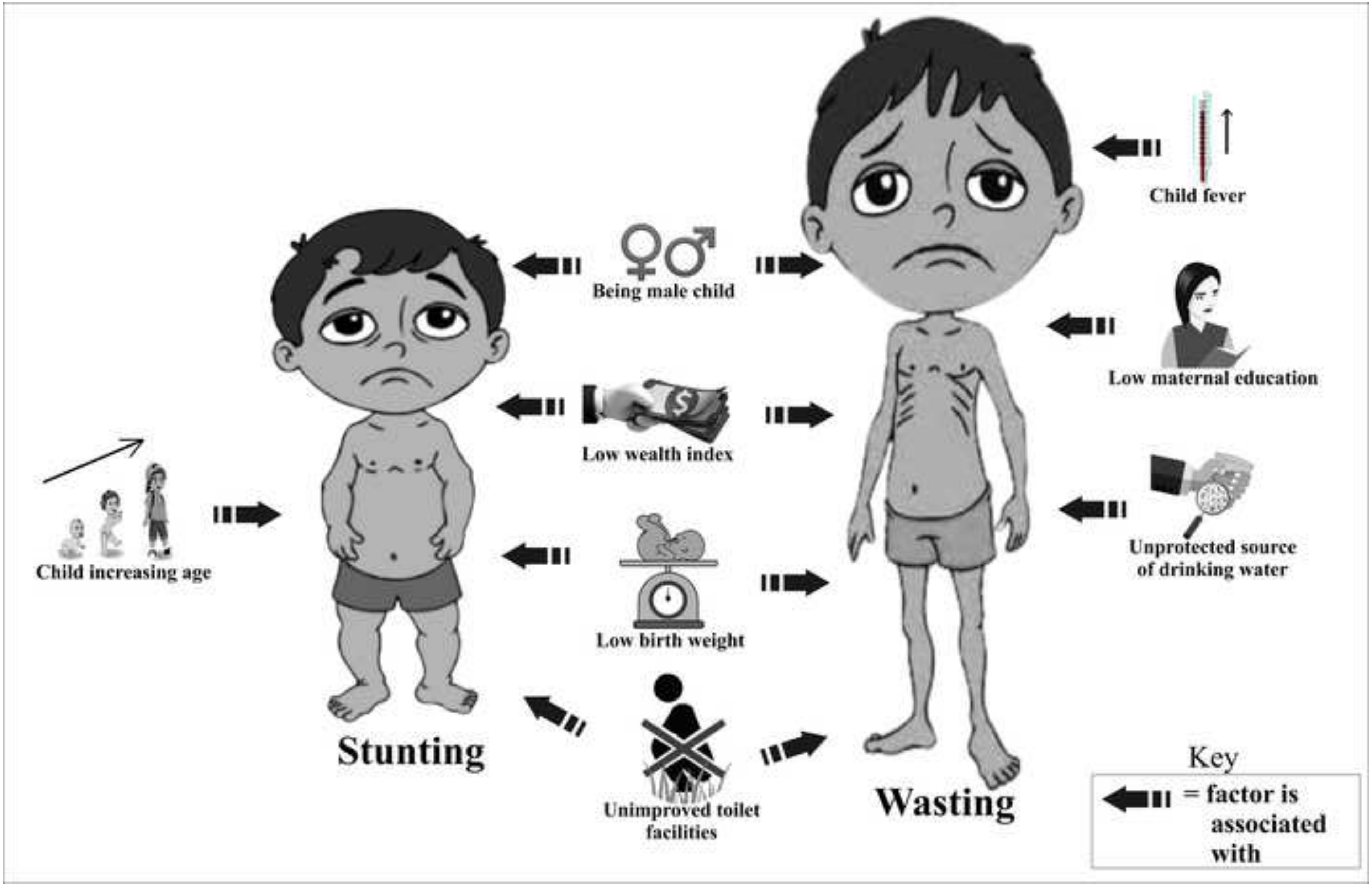

Click here to access/download
Supporting Information
STROBE checklist Ghana wasting and stunting.docx
