# Supplementary material for: Low birth weight, household socio-economic status, water and sanitation are associated with stunting and wasting among children aged 6–23 months: Results from a national survey in Ghana
Source: PLoS One. 2024 Mar 28;19(3):e0297698. doi: 10.1371/journal.pone.0297698 (PMC10977686; doi:10.1371/journal.pone.0297698)
Supplement: Attachment — Submitted filename: Respone to reviewers.docx [file pone.0297698.s003.docx]

Dear PLOS ONE Editor,

We are grateful to you and the reviewers for your time in improving our manuscript titled “Low birth weight, household socio-economic status, water and sanitation are associated with stunting and wasting among children aged 6-23 months: results from a national survey in Ghana” (PONE-D-23-13762). We have edited the manuscript to improve it as suggested by you and the reviewers. In the text following, we respond to each of the comments with references to the location in the main manuscript.

**Journal Requirements**

1. Please ensure that your manuscript meets PLOS ONE's style requirements, including those for file naming.
2. Data Availability statement
3. We note that Figure 2 in your submission contain copyrighted images.

**Authors’ response**

1. Please, Authors have made the necessary edits to the manuscript in conformity with PLOS ONE style requirements.
2. Data availability has been updated.
3. The Authors have not been successful at receiving feedback from the copyright holders. Although the figure was just for illustrative purposes only, we do acknowledge the ramifications moving forward. As such, Fig 2 and its caption on lines 356 and 357 has been expunged. Corresponding changes have therefore been made to line 351.

**Review #1 comment 1**

1. Firstly, it is important to clarify that this study represents a secondary analysis.

**Authors’ Response**

Thank you for drawing our attention to this very important detail. In the abstract section, on lines 34 to 36 we have replaced

“The 2017/18 Ghana Multi-Indicator Cluster Survey data was analysed” with

“This is a secondary data analysis of the most recent (2017/2018) Ghana Multi-Indicator Cluster Survey (MICS) datasets.”

And added the new statement to the Materials and methods section on lines 117 and 118.

**Review #1 comment 2**

Additionally, I would like to inquire if any statistical assessments were conducted to identify potential confounders and interactions.

**Authors’ Response**

We first conducted a bivariate analysis to identify the significant factors associated with the outcomes and all factors which were significantly associated with the outcome variables were included in the multivariate analysis to produce adjusted odds ratios. Further, we included pair-wise interaction effects of the independent variables, but none of them was statistically significant.

**Reviewer #2:**

Dear Authors,

I had the pleasure of reading your paper. It was an interesting read and indeed this topic is very important. My comments are as follows.

**Review #2 comment 1**

1) How did you choose the variables? there are several research papers on the same topic that use a long list of independent variables as determinants of stunting and wasting. How did you choose them? Did you follow any variable selection methodology?

**Authors’ response**

Thank you for committing your time to review this manuscript.

Inasmuch as Authors agree that there are several independent variables associated with stunting and wasting in literature. We also acknowledge that such variables are context-specific. It is important to say that this research was a secondary analysis of multi-indicator cluster survey datasets that were collected by the Ghana Statistical Service and other relevant stakeholders. The datasets contained a predefined and exhaustive list of health and nutrition-related variables including those that were included in this study.

As such, the inclusion of these variables was pre-defined before the conduct of this study by the primary data collectors. Additionally, after checking for the possible confounders from the literature, we conducted a bivariate analysis and included only variables that were statistically significant at p < 0.05 in the multivariate analyses. Our approach ensured we produced adjusted odds ratios for both outcomes modelled without including too many possible confounders which do not produce the model.

**Review #2 comment 2**

2) While it is interesting to have separate regressions for Stunting, wasting and one with double burden, I missed the relative interpretation of the results in the discussion. It would be nice to understand how results differ for two different types of undernutrition and what are the implications of those findings.

**Authors’ response**

For similarities in the determinants of both wasting and stunting and their implications, Authors have included the following in the Discussion section on lines 371 to 382.

“Our results suggest that male children have a higher odds of wasting and stunting and low birth weight increases the odds of stunting and wasting among children. Likewise, increasing household wealth reduces the odds of both wasting and stunting, emphasizing the role of poverty in malnutrition among children. Also, the results suggest that improving household sanitation reduces the odds of stunting and wasting among children. On the contrary, our results suggest that when the household head was male, the child had a higher odds of stunting, but this did not hold for wasting. Although we had no data on maternal empowerment index, our result may conform with the findings that female-headed households have better nutrition as women are more empowered to make decisions on the nutrition of the household [47], improving the nutrition of the household and children in the long-term. Further, our results suggest that low birth weight may have detrimental effects on the long-term nutrition of the child [48], as low birth weight was significantly associated with stunting but not wasting.”

**Review #2 comment 3**
3) Did you consider underweight as the third indicator of undernutrition as well? it would be nice to know your reasoning for excluding it from the analysis.

**Authors’ response**

The Authors decided to focus on wasting and stunting because they are more relevant in the public health and clinical care settings in Ghana. Stunting is the most prevalent of the 3 three forms of malnutrition (with 1 in 3 children being stunted) and the acute nature of wasting makes it very relevant in the in-patient care environment. Also, Authors are of the view that underweight is a composite measure of both stunting and wasting, addressing the determinants of these two indicators meant addressing underweight in a nutshell. I am sure it is for this same reason that the sustainable development Goal 2.2 focussed on only wasting and stunting. Concentrating on these 2 forms of undernutrition afforded Authors the opportunity to espouse a more focused narrative based on the study’s aims and scope.

Therefore for clarity, Authors added the below sentence to the Introduction, from lines 106 to 108.

“Limiting the outcome variables to only wasting and stunting is in tandem with the SDG [35] approach and mainly because underweight is a composite measure of both stunting and wasting.”

**Review #2 comment 4**

4) You have explained prevalence of stunting and wasting in the discussion session. Since these are your main variables, I expected these percentages at the beginning of the results section. It would be nice if you could move the discussion on stunting and wasting prevalence to the beginning of the results section.

**Authors’ response**

I appreciate your suggestions and have therefore moved the statement below from the Results section from line 276 to line 270.

“The prevalence of wasting and stunting among the children was 12.1% and 15.9% respectively.”

Authors are however unable to move the discussion of the prevalence of stunting and wasting from the discussion section to the Results section. This is due to the unique distinction between results and discussion sections as arranged by Authors.

Thank you, reviewer #2, for your patience.

All in-text suggestions for corrections have been effected were necessary, on lines 46, 79, 80, 106, 122, 123, 149 and 183. Additionally, specific changes have been made to the last row of Table 1 and 5^th^ column of Table 2 as recommended.

Thank you for your time.

Sincerely yours,

**Addae Yaw Hammond**

**Corresponding Author**
