# Supplementary material for: Low birth weight, household socio-economic status, water and sanitation are associated with stunting and wasting among children aged 6–23 months: Results from a national survey in Ghana
Source: PLoS One. 2024 Mar 28;19(3):e0297698. doi: 10.1371/journal.pone.0297698 (PMC10977686; doi:10.1371/journal.pone.0297698)
Supplement: Attachment — Submitted filename: Response to Reviewers.docx [file pone.0297698.s004.docx]

Dear PLOS ONE Editor,

We are grateful to you and the reviewers for your time in improving our manuscript titled “Low birth weight, household socio-economic status, water and sanitation are associated with stunting and wasting among children aged 6-23 months: results from a national survey in Ghana” (PONE-D-23-13762R1). The Authors have provided clarification on our statistical analyses and have provided references to justify the use of such analysis. We have also added more content to the discussion to fully address reviewer comments. In the following text, we provide further explanations to each of the comments with references to the location in the main manuscript.

**1. Review #2 comment 1**

I am fully aware that MICS has a predetermined set of variables for each round across countries. My concern was related to some commonly used variables that are available in MICS but missing from this analysis eg mother's media access, birth rank of the child, father's education, hand washing facility, agricultural land ownership etc. Why were these variables excluded?

Second paragraph of the author's response is much more problematic because I do not think that significance of bivariate correlations can be used for variable selection in multivariate analysis. Simplest methodology could be Backward Elimination starting from the most complete model. Current method is not correct and should be revised.

**Authors’ response**

Authors would want to kindly remind the reviewer that we did not use bivariate correlations in any of our analyses. In the instance where we used bivariate analysis, the authors used bivariate regression analyses to first identify the variables that were significant at 0.05 p-value or less. The use of these bivariate associations to justify a variable’s inclusion in a multivariate analysis is a rigorous approach to statistical analyses and has been justified and used extensively in literature.

The bivariate analysis is meant to serve as a screening tool, so only variables that produce the model will be included in the multivariate model. And this explains why some nutrition-related variables such as those listed by the reviewer may be present in the MICs dataset yet not included in our analysis. It becomes a challenge to include hundreds of variables in a multivariate model in the instance of a multi-indicator cluster survey. So, the need to first conduct this bivariate analysis before the multivariate.

Authors do however note the reviewer’s description of this approach as “problematic” and “not correct”. And would want to say that contrary to these statements, this approach is acceptable within this context. Due to its use in articles that have been published in this respected Journal and elsewhere, we are of the view that this approach is neither problematic nor incorrect.

For instance, in Abbas & Salman, 2023 (doi.org/10.1177/0192513X23120197) titled “*Women’s Attitudes Toward Intimate Partner Violence in Sindh, Pakistan: An Analysis of Multiple Indicators Cluster Survey”*, Abbas & Salman described the selection of their variables for inclusion in their multivariate analyses using this approach.

In another article that was published on a similar dataset in this Journal, Sakpota & Hu (2023) ([doi.org/10.1371/journal.pone.0287974](https://doi.org/10.1371/journal.pone.0287974)) in their analysis reported that *“First, bivariate analysis was conducted … to determine the p-value between diarrhea and independent variables, and variables having p-value less than 0.05 in bivariate analysis were considered candidate variables for the multilevel analysis”.*

Authors have therefore provided a book by Hosmer & Lemeshow (Applied Logistic Regression, 2004) as citation [43] in the statistical analysis section to buttress the rigour of this approach of selecting variables for inclusion in a multivariate regression model. In Hosmer & Lemeshow’s book, which has been cited more than 77,000 times, they provide further rationale for conducting a bivariate analysis before a multivariate analysis.

Please check line 230.

**2. Review #2 comment 2**

Thank you for adding the comparative analysis. Discussion on double burden is still missing.

**Authors’ response**

Thank you for drawing our attention to this. Authors have added the statement below to the discussion to cater for this omission.

“Contrary to another study [44], this present study’s finding implies female children were less likely than their male counterparts to be stunted and wasted. This is in tandem with previous studies on similar national datasets in Ghana [14,48] and other parts of Africa [49,50]. The increased susceptibility of male children to undernutrition might be due to the fact that male children are expected to grow slightly more rapidly, and the increased expected rate is perhaps more easily influenced by nutritional inadequacies and other exposures [51]. Also, children born low birth weight may already be lagging and disadvantaged in terms of their growth, making them prone to early developmental challenges including a compromised immune system and increased susceptibility to infections [52]. Increasing household wealth, on the other hand, reduces the odds of both wasting and stunting, emphasizing the role of poverty in malnutrition among children.”

Please check lines 364 to 376

**3. Review #2 comment 3**

While it is correct that underweight is a composite of stunting and wasting, the claim that it was excluded from SDGs for this reason needs a strong citation. Also, why does this reasoning disqualify underweight as an important indicator of undernutrition from your analysis?

**Authors’ response**

Thank you for requesting for more details on the reasons why Authors excluded underweight from the analysis in this present study. To provide further clarity Authors need to acknowledge the relevance of this study’s title and objectives. And that we followed and were guided by the title and objective of this study in conformity with established scientific literature.

The title of this manuscript reads. *“Low birth weight, household socio-economic status, water and sanitation are associated with* ***stunting and wasting*** *among children aged 6-23 months: results from a national survey in Ghana”.*

The objective of this study also reads*; “…purpose of this study was to investigate the relationship between IYCF indicators, socio-economic, demographic, water and sanitation factors, and the outcome variables stunting and wasting among children aged 6 to 23 months.”*

The objective and title were the overriding guide in conducting our analyses. And the title and objective as stated above are on wasting and stunting only. Moreover, the concentration of this study on these two indicators of malnutrition is an acceptable approach that conforms with established scientific literature.

For instance, studies published in this Journal have in the past concentrated on three indicators e.g. Boah et al., 2019 (doi.org/10.1371/journal.pone.0219665) or two indicators e.g. Mutanga et al., 2021 (doi.org/10.1371/journal.pone.0259765) or even one indicator e.g. Hossain et al., 2022 (doi.org/10.1371/journal.pone.0278097) based on their respective study titles and objectives.

In a similar context as this present study, Mutunga et al., in 2020 (doi.org/10.3390/nu12020559) analysed the MICs of 6 countries. In that study, Mutunga et al. focussed on only stunting and wasting. These references may indicate that, although commonly practised, it is not mandatory for a study to analyse all 3 z-score anthropometric indices of child undernutrition. It is permissible to select one or two indices to be included in a study depending on what the study’s objectives are.

**4. Review #2 comment 4**

I truly respect author's unique arrangement of arguments, but I am afraid uniqueness is not a good justification for any arrangement. Intuitive arrangement would explain the problem in detail (detailed analysis of prevalence of undernutrition) first and then move on to analyze the determinants of the problem. For me it is counterintuitive to discuss why a problem exists first and then discuss what the problem actually is.

**Authors’ response**

Authors perfectly agree with the reviewer that *“it is counterintuitive to discuss why a problem exists first and then discuss what the problem actually is.”* The Authors would kindly want to point out that from the first submission of this manuscript to the revised submission, the authors have not done anything different. We first discussed the prevalence of wasting and stunting in the initial paragraphs of the discussion from lines 353 to 363. And in the ensuing paragraphs, we discussed the determinants of stunting and wasting form lines 364 to 433.

What Authors disagreed with was the reviewer’s initial (first) review suggestion for Authors to *“…move the discussion on stunting and wasting prevalence to the beginning of the results section.”* For which reason Authors stated that the discussion and results sections are uniquely different.

We are thankful to the Editor and the reviewers for their meticulousness and very significant inputs, hopefully, this time this manuscript will be suitable for publication in this reputable Journal.

Sincerely yours

**Addae Yaw Hammond**

**Corresponding Authors**
